# Identification of Potential Key Genes Associated With the Pathogenesis and Prognosis of Gastric Cancer Based on Integrated Bioinformatics Analysis

**DOI:** 10.3389/fgene.2018.00265

**Published:** 2018-07-17

**Authors:** Xinkui Liu, Jiarui Wu, Dan Zhang, Zhitong Bing, Jinhui Tian, Mengwei Ni, Xiaomeng Zhang, Ziqi Meng, Shuyu Liu

**Affiliations:** ^1^Department of Clinical Chinese Pharmacy, School of Chinese Materia Medica, Beijing University of Chinese Medicine, Beijing, China; ^2^Evidence Based Medicine Center, School of Basic Medical Science, Lanzhou University, Lanzhou, China; ^3^Key Laboratory of Evidence Based Medicine and Knowledge Translation of Gansu Province, Lanzhou, China; ^4^Institute of Modern Physics, Chinese Academy of Sciences, Lanzhou, China

**Keywords:** gastric cancer, bioinformatics, differentially expressed genes, survival, biomarker, GEO, TCGA

## Abstract

**Background and Objective:** Despite striking advances in multimodality management, gastric cancer (GC) remains the third cause of cancer mortality globally and identifying novel diagnostic and prognostic biomarkers is urgently demanded. The study aimed to identify potential key genes associated with the pathogenesis and prognosis of GC.

**Methods:** Differentially expressed genes between GC and normal gastric tissue samples were screened by an integrated analysis of multiple gene expression profile datasets. Key genes related to the pathogenesis and prognosis of GC were identified by employing protein–protein interaction network and Cox proportional hazards model analyses.

**Results:** We identified nine hub genes (*TOP2A, COL1A1, COL1A2, NDC80, COL3A1, CDKN3, CEP55, TPX2*, and *TIMP1*) which might be tightly correlated with the pathogenesis of GC. A prognostic gene signature consisted of *CST2, AADAC, SERPINE1, COL8A1, SMPD3, ASPN, ITGBL1, MAP7D2*, and *PLEKHS1* was constructed with a good performance in predicting overall survivals.

**Conclusion:** The findings of this study would provide some directive significance for further investigating the diagnostic and prognostic biomarkers to facilitate the molecular targeting therapy of GC.

## Introduction

Although North America and most western European countries have seen a sharp decline in incidence and mortality over the past decades, gastric cancer (GC) remains the fifth most common malignancy worldwide and represents a serious medical burden especially in Eastern Asia (Ferro et al., 2014; Torre et al., 2015). In China, GC is the second most frequent cancer among males and the third among females, and is the second leading cause of cancer-related lethality in both males and females, which leads to an estimated 498,000 cancer deaths with about 679,000 newly diagnosed cancer cases in 2015 (Chen et al., 2016). Poor 5-year survival in GC is mainly attributed to the fact that most patients are diagnosed at an advanced stage and even with metastatic diseases and thus lose the opportunity for a curative resection (Cutsem et al., 2016; Zong et al., 2016; Li R. et al., 2017). Despite major advances in understanding the epidemiology, pathology, and molecular mechanisms of GC and in implementing emerging therapeutic options such as targeted and immune-based therapies, not all patients respond to existing molecularly targeted agents developed for certain acknowledged biomarkers (Ciliberto et al., 2015; Cutsem et al., 2016; Chau, 2017). Therefore, although biomarkers and therapeutic targets recently found in GC have made a great contribution to improving the diagnosis and treatment of GC, identifying novel diagnostic and prognostic biomarkers remains urgently necessary in terms of the biological complexity, poor prognosis and high reoccurrence of GC (Wadhwa et al., 2013; Cutsem et al., 2016; Wang et al., 2017; Kang et al., 2018).

In recent years, the advancement of microarray and high throughput sequencing technologies has provided an efficient tool to decipher critical genetic or epigenetic alternations in carcinogenesis and to discover promising biomarkers for cancer diagnosis, treatment and prognosis (Kulasingam and Diamandis, 2008; Cancer Genome Atlas Research Network, 2014). Meanwhile, in order to overcome the limited or inconsistent results due to the application of either different technological platforms or a small sample size, integrated bioinformatics methods have been adopted in cancer research and a vast range of valuable biological information has been uncovered (Yang et al., 2014; Song et al., 2017; Sun C. et al., 2017; Sun M. et al., 2017; Wang et al., 2017).

In the present study, we firstly performed an integrated analysis and identified differentially expressed genes (DEGs) by using microarray and RNA sequencing data in human GC and normal gastric tissue samples. Secondly, functional enrichment analysis was further conducted to analyze the main biological functions modulated by the DEGs. Finally, key genes affecting the pathogenesis and prognosis of GC patients were identified by utilizing protein–protein interaction (PPI) network and survival analyses.

## Materials and Methods

### Gene Expression Profile Data

Microarray data on gene expression (GSE19826, GSE27342, GSE29272, GSE33335, GSE54129, GSE56807, GSE63089, GSE65801, and GSE79973) were downloaded from Gene Expression Omnibus (GEO)^1^. All included datasets met the following criteria: (1) they employed human stomach tissue samples. (2) They contained case-control groups. (3) They contained at least ten samples. A large sample size may reliably reveal the DEGs or non-coding RNAs. The small sample size is reported to be one of the major challenges in microarray analysis, and recent integrated bioinformatics studies tend to use datasets with a relatively large sample size (Sun M. et al., 2017; Moradifard et al., 2018). Therefore, the GEO datasets which contained at least ten samples were chosen for further study. Raw RNA sequencing data containing 375 GC samples and 32 matched non-cancerous samples were obtained from The Cancer Genome Atlas (TCGA)^2^.

### Integrated Analysis of Microarray Datasets

Limma package (Ritchie et al., 2015) in R software was applied to perform the normalization and base-2 logarithm conversion for the matrix data of each GEO dataset, and the DEGs between tumor and normal tissues were also screened by the limma package. Gene integration for the DEGs identified from the nine datasets was conducted by an R package “RobustRankAggreg” (Kolde et al., 2012) based on a robust rank aggregation (RRA) method. This RRA method screens genes ranked consistently better than expected based on null hypothesis of uncorrelated inputs (Kolde et al., 2012). Thus, we did not integrate the gene expression values of samples from different datasets. And like many published papers based on the RobustRankAggreg package (Yang et al., 2014; Shi et al., 2015), we also did not perform batch effect correction. |log_2_FC| ≥ 1, *P*-value < 0.05 and adjust *P*-value < 0.05 were considered statistically significant for the DEGs.

### DEGs Validation by TCGA

The results of integrated analysis of GEO datasets were validated using the RNA sequencing data in the TCGA GC dataset. The data were normalized and analyzed by the edgeR package (Robinson et al., 2010). Genes with |log_2_FC| ≥ 1, *P*-value < 0.05 and adjust *P*-value < 0.05 were considered to be significantly differentially expressed. Overlapping DEGs between the integrated microarray and RNA sequencing data analyses were retained for further study. In addition, the normalized gene expression level of the TCGA GC dataset was transformed on the base-2 logarithm for further analysis.

### Functional Enrichment Analysis of DEGs

To elucidate potential biological processes, molecular functions and cellular components associated with the overlapping DEGs, we performed GO enrichment analysis utilizing the Database for Annotation, Visualization and Integrated Discovery (DAVID, version 6.8)^3^ (Huang da et al., 2009). And Kyoto Encyclopedia of Genes and Genomes (KEGG) pathway enrichment analysis was carried out by clusterProfiler (Yu et al., 2012) to expound promising signaling pathways correlated with the overlapping DEGs. *P*-value < 0.05 and adjust *P*-value < 0.05 were defined as the cut-off criteria.

### PPI Network and Module Analysis

The STRING (Szklarczyk et al., 2017) database was applied to identify potential interactions among the overlapping DEGs. PPIs with a confidence score ≥ 0.4 were reserved and further imported to Cytoscape (Shannon et al., 2003) for constructing the PPI network of overlapping DEGs. Moreover, to detect hub clustering modules in the PPI network, we performed module analysis utilizing Molecular Complex Detection (MCODE) (Bader and Hogue, 2003) app with default parameters in Cytoscape. GO and KEGG pathway enrichment analyses for significant modules were also made.

### Survival Analysis

The clinical information of patients with GC was also downloaded from TCGA. After removing patients without overall survival (OS) data and gene expression profiles of the overlapping DEGs, 368 patients with GC were used for survival analysis. Univariate Cox proportional hazards regression analysis was employed to identify candidate genes that were strongly correlated with survival. Then the candidate genes with *P*-value < 0.05 were further applied in multivariate Cox proportional hazards regression analysis to identify prognostic gene markers. Subsequently, these prognostic gene markers were fitted in a multivariate Cox proportional hazards regression model with OS as a dependent variable to estimate their relative contributions to survival prediction. We constructed a prognostic gene signature according to a linear combination of gene expression values multiplied by a regression coefficient (*β*) accessed from the multivariate Cox proportional hazards regression model of each gene. The formula is as follows: risk score = expression of gene_1_ × *β*_1_gene_1_ + expression of gene_2_ × *β*_2_gene_2_ + … expression of gene_n_ × *β*_n_gene_n_ (Zhou et al., 2015; Xin et al., 2016; Huang et al., 2017). These GC patients were classified into either low- or high-risk groups based on the median prognostic risk score. Furthermore, we performed time-dependent receiver operating characteristic (ROC) curve analysis by employing an R package “survivalROC” to assess the predictive accuracy of the prognostic signature for time-dependent cancer death (Heagerty and Zheng, 2005). The area under the curve (AUC) was calculated to measure the predictive ability of the gene signature for clinical outcomes.

### Statistical Analysis

The univariate and multivariate Cox proportional hazards regression analyses were conducted utilizing an R package “survival”. Hazard ratio (HR) and 95% confidence interval (CI) were calculated to identify protective (HR < 1) or risky genes (HR > 1). A survival curve made by Kaplan–Meier method was implemented to estimate the differences in survival time between the high- and low-risk patients. All the statistical analyses were conducted with R (version 3.4.3)^4^.

## Results

### Identification of DEGs

**FIGURE 1:**
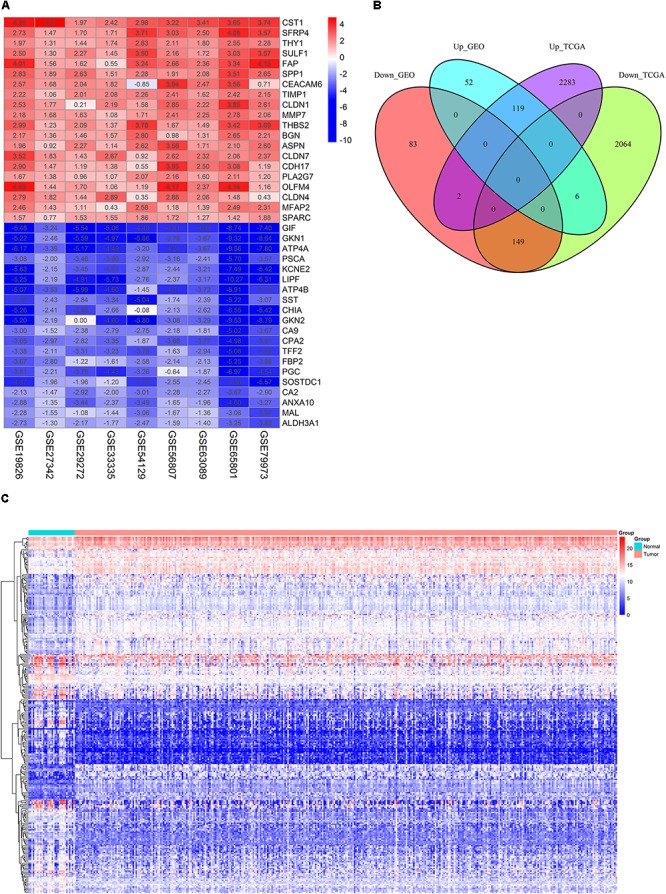
Identification of DEGs. **(A)** The heat map of top 20 down- and up-regulated DEGs in the integrated microarray analysis. Each column represents one dataset and each row represents one gene. The number in each rectangle represents the value of log_2_FC. The gradual color ranging from blue to red represents the changing process from down- to up-regulation. **(B)** Venn diagrams of the DEGs between the integrated nine GEO datasets and the TCGA GC dataset. **(C)** The heat map of 268 overlapping DEGs in GC and normal gastric tissues (TCGA dataset). Each column represents one sample and each row represents one gene. The gradual color ranging from blue to red represents the changing process from down- to up-regulation.

The detailed information for the samples in the included datasets was shown in Supplementary Table 1. The information for the nine GEO datasets included in the current study was displayed in **Table 1**. A total of 411 DEGs comprising 234 down-regulated and 177 up-regulated genes were obtained after the integrated analysis of nine GEO datasets (Supplementary Table 2). **Figure 1A** showed top 20 down- and up-regulated genes in the integrated microarray analysis. The DEGs acquired from the TCGA GC dataset consisted of 2219 down-regulated and 2404 up-regulated genes (Supplementary Table 3). We further identified 268 overlapping DEGs (149 down-regulated and 119 up-regulated genes) by intersecting the results of integrated microarray and RNA sequencing data analyses (**Figures 1B,C** and Supplementary Table 4).

**Table 1:** Information for the nine GEO datasets included in the current study.

Dataset	Reference	Platform	Number of samples (Tumor/Control)
GSE19826	Wang et al., 2012	[HG-U133_Plus_2] Affymetrix Human Genome U133 Plus 2.0 Array	27 (12/15)
GSE27342	Cui et al., 2011a,b	[HuEx-1_0-st] Affymetrix Human Exon 1.0 ST Array [transcript (gene) version]	160 (80/80)
GSE29272	Wang et al., 2013; Li et al., 2014	[HG-U133A] Affymetrix Human Genome U133A Array	268 (134/134)
GSE33335	Cheng et al., 2012a,b, 2013	[HuEx-1_0-st] Affymetrix Human Exon 1.0 ST Array [transcript (gene) version]	50 (25/25)
GSE54129	Sun C. et al., 2017	[HG-U133_Plus_2] Affymetrix Human Genome U133 Plus 2.0 Array	132 (111/21)
GSE56807	Wang et al., 2014	[HuEx-1_0-st] Affymetrix Human Exon 1.0 ST Array [transcript (gene) version]	10 (5/5)
GSE63089	Zhang et al., 2015	[HuEx-1_0-st] Affymetrix Human Exon 1.0 ST Array [transcript (gene) version]	90 (45/45)
GSE65801	Li H. et al., 2015	Agilent-028004 SurePrint G3 Human GE 8x60K Microarray (Probe Name Version)	64 (32/32)
GSE79973	He et al., 2016	[HG-U133_Plus_2] Affymetrix Human Genome U133 Plus 2.0 Array	20 (10/10)


### Functional Enrichment Analysis of DEGs

**FIGURE 2:**
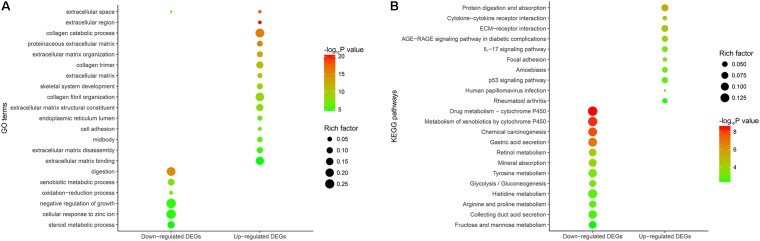
Functional enrichment analysis of the overlapping DEGs. **(A)** GO enrichment analysis of the overlapping DEGs. The *y*-axis shows significantly enriched GO terms, and the *x*-axis shows different gene categories. Rich factor refers to the ratio of the number of DEGs enriched in a GO term to the number of all the annotated genes enriched in the GO term. **(B)** KEGG pathway enrichment analysis of the overlapping DEGs. The *y*-axis shows significantly enriched KEGG pathways, and the *x*-axis shows different gene categories. Rich factor refers to the ratio of the number of DEGs enriched in a KEGG pathway to the number of all the annotated genes enriched in the KEGG pathway.

We conducted GO and KEGG pathway enrichment analyses to expound the potential biological functions of 268 DEGs. In terms of the 149 down-regulated genes, they were significantly enriched in multiple biological processes related to metabolism (**Figure 2A** and Supplementary Table 5). As for the 119 up-regulated genes, they showed a close correlation with extracellular matrix, such as extracellular matrix organization, extracellular matrix disassembly, extracellular matrix structural constituent and so on. (**Figure 2A** and Supplementary Table 5).

According to KEGG pathway enrichment analysis, the down-regulated genes mainly participated in diverse metabolism-associated signaling pathways, like drug metabolism – cytochrome P450, metabolism of xenobiotics by cytochrome P450, retinol metabolism, tyrosine metabolism and so on (**Figure 2B** and Supplementary Table 6). As for the up-regulated genes, they mainly regulated pathways correlated with environmental information processing and tumor progression, such as cytokine-cytokine receptor interaction, ECM-receptor interaction, focal adhesion and so on (**Figure 2B** and Supplementary Table 6).

### PPI Network and Module Analysis

**FIGURE 3:**
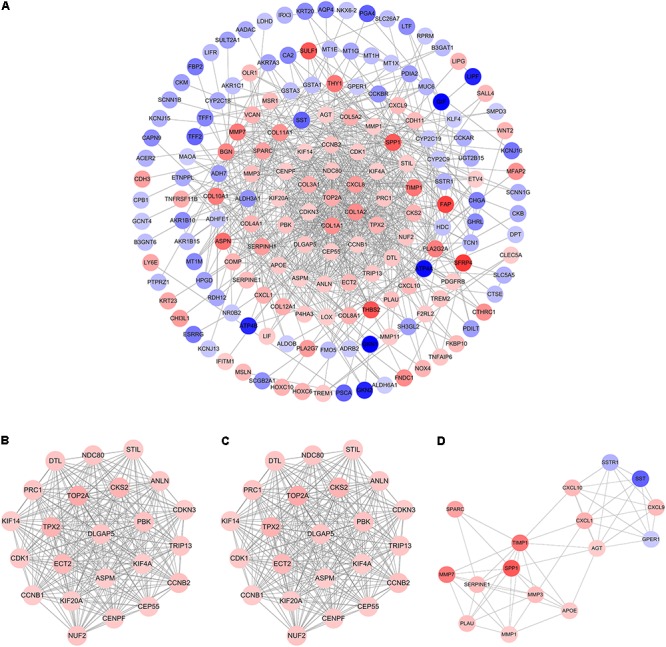
Protein–protein interaction (PPI) network and hub clustering modules. **(A)** The PPI network of overlapping DEGs. **(B)** Module 1 (MCODE score = 22.818). **(C)** Module 2 (MCODE score = 10.8). **(D)** Module 3 (MCODE score = 7.467). Blue circles represent down-regulated genes and red circles represent up-regulated genes. Node color deepens as the value of |log_2_FC| increases.

**FIGURE 4:**
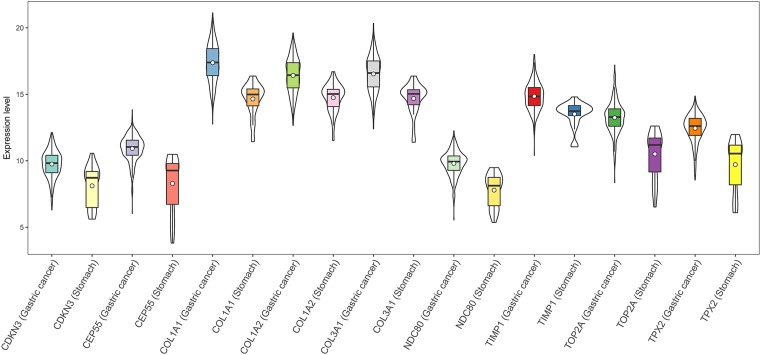
Expression of the nine hub DEGs in GC and normal gastric tissues (TCGA dataset). Expression values of genes are log_2_-transformed.

**FIGURE 5:**
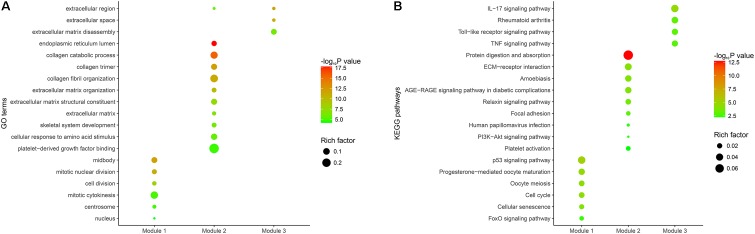
Functional enrichment analysis of the DEGs in the three modules. **(A)** GO enrichment analysis of the DEGs in the three modules. The *y*-axis shows significantly enriched GO terms, and the *x*-axis shows different modules. Rich factor refers to the ratio of the number of DEGs enriched in a GO term to the number of all the annotated genes enriched in the GO term. **(B)** KEGG pathway enrichment analysis of the DEGs in the three modules. The *y*-axis shows significantly enriched KEGG pathways, and the *x*-axis shows different gene categories. Rich factor refers to the ratio of the number of DEGs enriched in a KEGG pathway to the number of all the annotated genes enriched in the KEGG pathway.

The PPI network of overlapping DEGs consisted of 173 nodes and 711 interactions (**Figure 3A** and Supplementary Table 7). Two topological features, degree (Williams and Del Genio, 2014) and betweenness (Newman, 2005) were calculated to identify candidate hub nodes. The higher the two quantitative values of a gene, the more important it is in this network. As a result, 10 candidate hub nodes, the degree and betweenness of which were all more than four-fold of the corresponding median values, were identified, namely, DNA topoisomerase II alpha (*TOP2A*), collagen type I alpha 1 chain (*COL1A1*), collagen type I alpha 2 chain (*COL1A2*), C-X-C motif chemokine ligand 8 (*CXCL8*), NDC80 kinetochore complex component (*NDC80*), collagen type III alpha 1 chain (*COL3A1*), cyclin dependent kinase inhibitor 3 (*CDKN3*), centrosomal protein 55 (*CEP55*), TPX2 microtubule nucleation factor (*TPX2*), and TIMP metallopeptidase inhibitor 1 (*TIMP1*) (Supplementary Table 8). Additionally, in order to detect significant clustering modules in this PPI network we performed module analysis and obtained top three modules with high scores (**Figures 3B–D**). The nine candidate hub nodes except *CXCL8* were contained in the three modules, which implied that the three modules might remarkably represent the key biological characteristics of this PPI network, and thereby the nine nodes were defined as major hub nodes in the PPI network (**Figure 4**). At the aspect of GO enrichment analysis, module 1 was closely correlated with mitotic nuclear division, cell division, mitotic cytokinesis, midbody, centrosome, and nucleus; module 2 was highly connected to collagen catabolic process, collagen fibril organization, extracellular matrix structural constituent, platelet-derived growth factor binding, endoplasmic reticulum lumen, and collagen trimer; module 3 was intimately associated with extracellular matrix disassembly, extracellular region, and extracellular space (**Figure 5A** and Supplementary Table 9). With respect to KEGG pathway enrichment analysis, the genes in module 1 were mainly enriched in p53 signaling pathway, cell cycle, and FoxO signaling pathway; the genes in module 2 mainly participated in ECM-receptor interaction, focal adhesion, and PI3K-Akt signaling pathway; the genes in module 3 were mainly implicated in Toll-like receptor signaling pathway and TNF signaling pathway (**Figure 5B**, Supplementary Table 10). Our data presented that once some DEGs were overexpressed the signaling pathways that they involved in may be dysregulated. For instance, highly up-regulated COL1A2, COL1A1, and COL4A1 in GC tissues might be responsible for the dysfunction of ECM-receptor interaction, focal adhesion, and PI3K-Akt signaling pathway; SPP1, CXCL10, and CXCL9 in Toll-like receptor signaling pathway were overexpressed as well. Since the three down-regulated genes (SSTR1, SST, and GPER1) in module 3 cannot be significantly enriched in any KEGG pathways identified in module analysis, all these KEGG pathways were enriched by the up-regulated genes in the three modules. And of the three down-regulated genes, only SSTR1 was significantly enriched in GO terms (extracellular region and extracellular space) identified in module analysis.

### Survival Analysis

**FIGURE 6:**
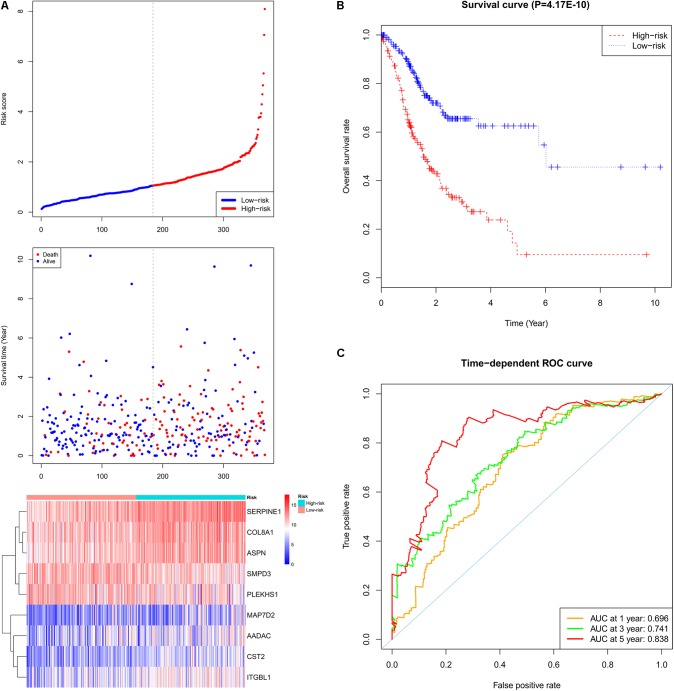
Prognostic gene signature of the nine genes in the GC patients (TCGA dataset). **(A)** From top to bottom is the risk score distribution, patients’ survival status distribution, and the heat map of the nine genes for low- and high-risk groups. In the heat map, each column represents one sample and each row represents one gene, and the gradual color ranging from blue to red represents the changing process from down- to up-regulation. **(B)** The Kaplan–Meier curves for low- and high-risk groups. **(C)** The ROC curves for predicting OS in GC patients by the risk score.

**FIGURE 7:**
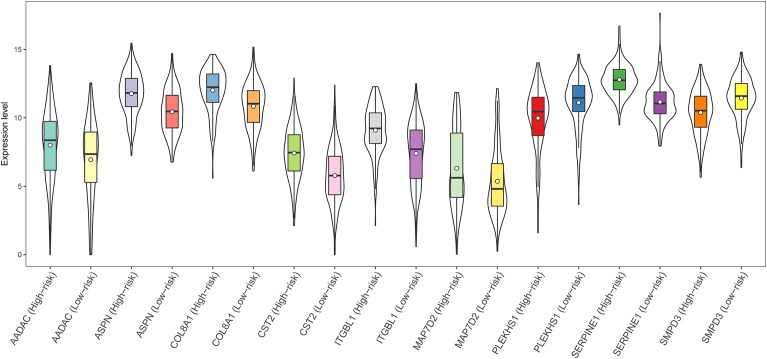
Expression of the nine genes in low- and high-risk groups (TCGA dataset). Expression values of genes are log_2_-transformed.

A total of 44 genes significantly correlated with survival time (*P* < 0.05) were identified by the univariate Cox proportional hazards regression model (Supplementary Table 11). A prognostic gene signature composed of nine genes was developed after using the multivariate Cox proportional hazards regression model, including cystatin SA (*CST2*), arylacetamide deacetylase (*AADAC*), serpin family E member 1 (*SERPINE1*), collagen type VIII alpha 1 chain (*COL8A1*), sphingomyelin phosphodiesterase 3 (*SMPD3*), asporin (*ASPN*), integrin subunit beta like 1 (*ITGBL1*), microtubule-associated protein 7 domain containing 2 (*MAP7D2*), and pleckstrin homology domain containing S1 (*PLEKHS1*) (**Table 2**). Among these nine genes, *COL8A1, SMPD3*, and *PLEKHS1* with HR < 1 were identified as protective prognostic genes, whereas *CST2, AADAC, SERPINE1, ASPN, ITGBL1*, and *MAP7D2* with HR > 1 were identified as risky prognostic genes. A total of 184 patients with the risk scores larger than the median risk score (1.060) were divided into the high-risk group, whereas the other 184 patients were divided into the low-risk group. The risk score result of the TCGA GC dataset was presented in **Figure 6A**. As shown in **Figure 6B**, a highly significant difference in OS was detected between the high- and low-risk groups (*P* < 0.0001). In details, the OS rate of patients in the low-risk group was 88.3% (95% CI = 83.50–93.40%), 65.5% (95% CI = 57.20–75.00%) and 62.5% (95% CI = 53.10–73.60%) for 1-, 3-, and 5-year, respectively, compared with 64.70% (95% CI = 57.65–72.60%), 31.25% (95% CI = 23.37–41.80%), and 9.52% (95% CI = 2.99–30.30%) in the high-risk group. The prognostic gene signature presented a good performance in survival prediction, as the AUC was 0.696, 0.741, and 0.838 for 1-, 3-, and 5-year OSs (**Figure 6C**), respectively. The expression level distribution of the nine genes in low- and high-risk groups was shown in **Figure 7**.

**Table 2:** Prognostic value of the nine genes in the GC patients of the TCGA cohort.

Gene symbol	Univariate analysis		Multivariate analysis		
		
	HR (95% CI)	*P*-value	HR (95% CI)	*P*-value	Coefficient
CST2	1.113 (1.029–1.204)	0.0075	1.083 (0.975–1.203)	0.1359	0.0797
AADAC	1.074 (1.009–1.143)	0.0244	1.112 (1.042–1.186)	0.0015	0.1058
SERPINE1	1.261 (1.133–1.403)	<0.0001	1.358 (1.179–1.564)	<0.0001	0.306
COL8A1	1.115 (1.011–1.230)	0.0289	0.612 (0.470–0.797)	0.0003	-0.4913
SMPD3	0.883 (0.803–0.971)	0.0100	0.917 (0.815–1.031)	0.1460	-0.0871
ASPN	1.143 (1.038–1.258)	0.0063	1.296 (1.090–1.542)	0.0034	0.2596
ITGBL1	1.105 (1.025–1.192)	0.0092	1.209 (1.042–1.404)	0.0126	0.1901
MAP7D2	1.068 (1.008–1.132)	0.0268	1.064 (1.000–1.133)	0.0512	0.0624
PLEKHS1	0.912 (0.846–0.982)	0.0148	0.937 (0.859–1.023)	0.1466	-0.0646


## Discussion

Integrated bioinformatics analysis mainly focusing on differentially expressed molecule screen, network-based hub node discovery, and survival analysis has been extensively applied to identify potential biomarkers associated with the diagnosis, treatment, and prognosis of GC. For example, Chang et al identified hub genes related to liver metastasis of GC from four GEO datasets by developing an integrated method including DEG screen, pathway analysis, literature-based annotations, PPI networks, reverse transcription-quantitative polymerase chain reaction (RT-qPCR), and immunohistochemistry (Chang et al., 2009); Sun et al identified key genes in the occurrence and development of GC from one GEO dataset using a bioinformatics approach incorporating DEG screen, functional enrichment analysis, PPI network construction, and survival analysis (Sun C. et al., 2017); Li X. et al. (2017) identified candidate biomarkers for GC from six GEO datasets by performing DEG, gene functional enrichment, and PPI network analyses, and validated their results with RT-qPCR; Ren et al. (2017) identified key genes and pathways for GC by a network-based method that combined data on gene expression, miRNA expression, DNA methylation, and DNA copy number in TCGA; Wang et al. (2017) used the gene expression profiles from one GEO dataset and TCGA, and identified a prognostic gene signature for predicting the survival of GC patients by a robust likelihood-based survival model. Compared with previous works, the current study not only integrated microarray data with relative large sample size from multiple GEO datasets and RNA sequencing data from TCGA, but also built gene networks and a Cox proportional hazards model to identify potential diagnostic and prognostic biomarkers in GC.

In the present study, nine microarray datasets were integrated with RNA sequencing data from TCGA, and 268 DEGs between GC and normal samples were identified, comprising 149 down-regulated and 119 up-regulated genes. The functional enrichment analysis showed that the down-regulated genes were primarily implicated in various metabolic processes, including metabolism of xenobiotics, cofactors, vitamins, amino acids, and carbohydrates. For the up-regulated genes, they mainly played important functions in signal transduction, cell growth and death, infectious diseases, and immune system. Particularly, many up-regulated genes were enriched in cancer-related pathways, such as ECM-receptor interaction, PI3K-Akt signaling pathway, and Toll-like receptor signaling pathway, which suggested these genes might be important in carcinogenesis and metastasis of GC. Our findings in the functional enrichment analysis agreed with previous works (Li H. et al., 2015; Li X. et al., 2017; Ren et al., 2017; Sun C. et al., 2017).

We also identified nine major hub genes in the PPI network, namely, *TOP2A, COL1A1, COL1A2, NDC80, COL3A1, CDKN3, CEP55, TPX2* and *TIMP1*, and coincidentally all of them were up-regulated genes in GC. The alteration of *TOP2A* in gene copy number and gene expression level is usually found in cancer cells, and deregulation of TOP2A expression might play an important role in chromosome instability and tumorigenesis (Chen et al., 2015). Moreover, highly expressed TOP2A enhances the risk of hematogenous recurrence in patients with stage II/III GC (Terashima et al., 2017). COL1A1 and COL1A2 are among the type I collagen family members which are widely believed to participate in carcinogenesis (Ramaswamy et al., 2003; Wolf et al., 2009). Overexpression of COL1A1 and COL1A2 has been confirmed in GC (Li et al., 2016; Sun, 2016; Zhuo et al., 2016; Wang and Yu, 2018) and may predict an adverse prognosis in GC patients (Li et al., 2016). Recent evidence showed that miR-129-5p could inhibit the proliferation, invasion, and migration of GC cells by selectively decreasing the expression of COL1A1 (Wang and Yu, 2018). Furthermore, *COL1A2* gene silencing was recently reported to suppress GC cell proliferation, invasion, and migration while facilitating apoptosis via deactivating PI3k-Akt signaling pathway (Ao et al., 2018). *COL3A1*, a member of type III collagen gene family, was regarded as a potential important gene in human GC using bioinformatics approaches (Hu and Chen, 2012; Chen et al., 2017). Nevertheless, investigations on the regulatory mechanism of COL3A1 in GC have been rarely reported. The mRNA and protein levels of NDC80 (also called HEC1), a member of the NDC80 complex, are commonly overexpressed in several human cancers including GC (Qu et al., 2014). NDC80 exerts significant functions in maintaining GC cell growth *in vitro* and *in vivo*, and high NDC80 expression might occur at the early stage of GC (Qu et al., 2014). CDKN3 has been proposed as a potential therapeutic target for GC and plays pivotal roles in the tumorigenesis of GC (Li Y. et al., 2017). Specifically, increased CDKN3 expression is frequently observed in GC tissues and cell lines and has a close correlation with advanced clinical stage, recurrence, and an adverse prognosis in GC (Li Y. et al., 2017). Besides, downregulation of CDKN3 could not only inhibit proliferation, invasion, and migration in GC, but also induce cell cycle arrest and apoptosis (Li Y. et al., 2017). Strongly elevated expression of CEP55 is detected in GC tissues and cell lines and shows a high correlation with the proliferation, colony formation and tumorigenesis of GC cells (Tao et al., 2014). Additionally, knockdown of CEP55 possibly suppressed proliferation in GC by inducing cell cycle arrest at G2/M phase (Tao et al., 2014). It has been demonstrated that TPX2 is overexpressed in multiple malignancies including GC, and high TPX2 expression is reported to be relevant to GC progression and might act as a potential indicator for a poor prognosis in GC patients (Liang et al., 2016; Shao et al., 2016; Tomii et al., 2017). The prognostic value of TIMP1 as a biomarker in GC is controversial, and its role in tumor invasion and metastasis seems fairly complicated although TIMP1 functions as an inhibitor of matrix metalloproteinases which are highly expressed in cancer and promote tumor invasion and the development of metastatic disease (Bao et al., 2010; Grunnet et al., 2013). A study based on literature search revealed that increased protein levels of TIMP1 in either tumor tissue extracts or in plasma from GC patients have a correlation with adverse outcomes (Grunnet et al., 2013). Moreover, recent findings showed that tumor-related myofibroblasts are the major source of elevated TIMP1 expression in GC (Alpizar-Alpizar et al., 2016).

The current study identified nine pivotal genes associated with GC prognosis and constructed a prognostic gene signature comprised of these genes. As for the three protective prognostic genes (*COL8A1, SMPD3*, and *PLEKHS1*), the prognostic value of *COL8A1* in GC has been evaluated before. COL8A1 might involve in the proliferation, adherence and migration of diverse cells, and overexpressed COL8A1 is detected in several rapidly proliferating cells, such as epithelial cells and tumor cells (Paulus et al., 1991; Bendeck et al., 1996; Xu et al., 2001; Tanaka and Arii, 2006; Wang et al., 2017). And the association of COL8A1 with multiple tumors has gained widely attention. For example, it was reported that down-regulation of *COL8A1* could obviously inhibit the proliferation and colony formation of hepatocarcinoma cells (Zhao et al., 2009). Moreover, a latest study based on co-expression network analysis observed that overexpression of COL8A1 is relevant to the adverse prognosis of human colon adenocarcinoma (Shang et al., 2018). Likewise, high expression of *COL8A1* also indicated poor clinical outcomes in GC according to the prognostic gene signature model built by Wang et al. (2017). However, unlike the earlier study, our prognostic model was based on the genes commonly identified as DEGs in multiple distinct datasets, which may account for the different results. Even so, future studies are warranted to validate our results. The prognostic value of *SMPD3* and *PLEKHS1* in GC has not been validated in previous studies. *SMPD3* encodes neutral sphingomyelinase-2 (nSMase2), a sphingomyelinase that catalyzes the hydrolysis of sphingomyelin in biological membranes to ceramide and phosphorylcholine (Wang et al., 2015). *SMPD3* as a potential tumor suppressor gene has gained widely studies, and it is linked to numerous malignancies like leukemia, breast cancer, and liver cancer (Bhati et al., 2008; Kim et al., 2008; Singh et al., 2014; Zhong et al., 2018). Also, abnormal promoter methylation of *SMPD3* has been reported in breast cancer, colorectal cancer, clear cell renal cell carcinoma, and hepatocellular carcinoma cells (Demircan et al., 2009; Shen et al., 2012; Revill et al., 2013; Wang et al., 2015). *PLEKHS1* remains a largely uncharacterized gene (Weinhold et al., 2014; Kotoh et al., 2016). Mutations in non-coding regions of *PLEKHS1* were found in cancer patients according to a genome-wide analysis (Weinhold et al., 2014). Furthermore, *Plekhs1* was identified as a potential contributor to mild hyperglycemia relevant to obesity in a rat model (Kotoh et al., 2016). Although the correlation between these three genes and GC has not been absolutely clarified and further studies are still demanded to validate our findings, the importance of these three genes as basic elements in the nine-gene signature should not be underestimated.

With regard to the six risky prognostic genes (*CST2, AADAC, SERPINE1, ASPN, ITGBL1*, and *MAP7D2*), the correlation of *CST2, SERPINE1, ASPN*, and *ITGBL1* with GC has been investigated before. *CST2* gene encodes Cystatin SA, which is among cystatin (CST) superfamily members functioning as cysteine protease inhibitors (Dai et al., 2017). Cystatins are proven to play a key part in tumor invasion and metastasis (Hirai et al., 1999; Nishikawa et al., 2004; Saleh et al., 2005; Dai et al., 2017). Similarly, it is found that high expression of salivary cystatin CST2 could promote *in vivo* bone metastasis (Blanco et al., 2012). In addition, the prognostic gene signature model made by Wang et al. (2017) also identified elevated *CST2* expression as an unfavorable predictor for clinical outcomes in GC. *SERPINE1* encodes plasminogen activator inhibitor 1 (PAI-1), and PAI-1 as a serine protease inhibitor exerts a critical role in the plasminogen-plasmin system owing to its function of inhibiting tissue-type and urokinase-type plasminogen activators (Declerck and Gils, 2013). PAI-1 has been known as a poor prognostic factor in several common tumors, and is involved in the invasion, metastasis, and the apoptosis inhibition of multiple tumor cells (Schmitt et al., 1997; Kwaan et al., 2000; Rømer et al., 2005; Fang et al., 2012). It is found that miR-30b might facilitate apoptosis and inhibit tumor growth by suppressing PAI-1 expression in GC (Zhu et al., 2014). Furthermore, an investigation based on DNA microarray indicated that overexpression of PAI-1 is correlated with aggressive lymph node metastasis in advanced GC (Suh et al., 2015). ASPN belongs to a family of small leucine-rich proteoglycans (Nakajima et al., 2007), and it is known as a major component of tumor stroma and its aberrant expression has been found in multiple tumors (Turashvili et al., 2007; Turtoi et al., 2011; Klee et al., 2012; Ansari et al., 2014). It has been reported that ASPN and other related matrix proteoglycans are correlated with the tumorigenesis and development of human GC (Theocharis et al., 2003; Wang et al., 2011; Hu et al., 2014; Satoyoshi et al., 2015). Additionally, overexpressed ASPN promotes the progression and metastasis of GC by regulating the epidermal growth factor receptor (EGFR) signaling pathway (Ding et al., 2015). *ITGBL1* gene encodes a beta integrin-related extracellular matrix protein called integrin beta-like protein 1 (Li R. et al., 2017). ITGBL1 contains 10 EGF-like repeats domain and is remarkably similar to integrin beta subunits (Berg et al., 1999). Existing studies presented that highly expressed ITGBL1 facilitates breast cancer bone metastasis and ovarian cancer cell migration and adhesion (Li X.Q. et al., 2015; Sun et al., 2016), while down-regulated ITGBL1 promotes cell invasion in non-small cell lung cancer (Gan et al., 2016). Moreover, recent evidence suggested that elevated ITGBL1 predicts adverse clinical outcomes in GC and might implicate the invasion and metastasis of GC cells by inducing epithelial-mesenchymal transition (Li R. et al., 2017). To sum up, the consistency between our findings and the results in previous studies confirms the reliability of our data analysis approaches. In terms of *AADAC* and *MAP7D2*, little is known about their prognostic value in GC. AADAC is a major serine esterase that extensively implicates the hydrolysis of diverse clinical drugs, and it is highly expressed in human liver and gastrointestinal tract (Kobayashi et al., 2012; Yoshida et al., 2018). MAP7D2 belongs to the MAP7 family of microtubule-associated proteins (Koizumi et al., 2017). MAPs play a major role in numerous critical cellular and intracellular activities, such as cell division, motility, differentiation and so on (Bhat and Setaluri, 2007). High expression of MAP7 predicts the tumor recurrence and adverse outcomes in colon cancer and is related to a poor prognosis in patients with cytogenetically normal acute myeloid leukemia (Blum et al., 2008; Fu et al., 2016). Although the status of *AADAC* and *MAP7D2* and their correlation with prognosis in GC have seldom been reported in the findings from earlier works, they could provide helpful evidence for potential prognostic biomarkers in future studies due to their significance in the nine-gene signature model.

The limitations of our study were as follows: (1) biological experiments are urgently demanded to validate our results because our study was performed based on data analysis; (2) the data used in this study were accessed from publicly available databases and we cannot evaluate the quality of these data; (3) the characteristic details (for example, gender, age, race, tumor grade and stage, *etc*.) were not taken into account since our study merely focused on the genes commonly identified as significantly altered ones in multiple datasets. Therefore, some biological information may be overlooked in our study.

## Conclusion

In conclusion, with the employment of multiple gene expression profile datasets and integrated bioinformatics analysis, we identified nine hub genes which might be involved in the pathogenesis of GC. Besides, a nine-gene signature which might act as a potential prognostic biomarker in patients with GC was constructed, and the prognostic model presented a good performance in predicting 1-, 3-, and 5-year OSs. These findings would provide some directive significance for the future prognosis prediction and molecular targeting therapy of GC. However, further experimental studies are urgently demanded to validate our results because our study was performed based on data analysis.

## Author Contributions

XL: conception, design, and performance of the research and writing of the paper. JW: supervision of the research. ZB and JT: provision of useful suggestions in methodology. DZ, MN, XZ, ZM, and SL: provision of suggestions in figure preparation. All authors read and approved the final version of the manuscript.

## Conflict of Interest Statement

The authors declare that the research was conducted in the absence of any commercial or financial relationships that could be construed as a potential conflict of interest.

## Footnotes

1 http://www.ncbi.nlm.nih.gov/geo/

2 https://cancergenome.nih.gov/

3 https://david.ncifcrf.gov/

4 https://www.r-project.org/
